# Atherosclerosis, Inflammation, and Genetics - And you Thought it Was Just LDL-cholesterol

**DOI:** 10.36660/abc.20200038

**Published:** 2020-02

**Authors:** Luis Henrique Wolff Gowdak

**Affiliations:** Universidade de São Paulo (USP), Faculdade de Medicina do Hospital das Clínicas do Instituto do Coração (Incor), São Paulo, SP - Brazil

**Keywords:** Atherosclerosis/physiopathology, Inflammation, Coronary Artery Disease/history, Mortality and Morbidity, Polymorphism, Genetic, Risk Factors

As the twentieth century unfolded, based on numerous epidemiological observations and intervention trials, cardiovascular risk factors were identified and targeted with the aim of decreasing cardiovascular disease burden worldwide. Along the centuries, changes in human eating patterns, a progressive decrease in physical activity, and a higher prevalence of obesity, all of which are contributing factors to the alarming rates of diabetes, hypertension, and hypercholesterolemia we see in our daily practice, have led us to assume that atherosclerosis-related disorders (myocardial infarction, ischemic cardiomyopathy, stroke, and peripheral artery disease) are an inevitable consequence of the evolutionary process we have to face in present times. However, due to advances in noninvasive imaging of the vascular system, atherosclerotic lesions in the aorta and coronary and carotid arteries happen to be found in mummies from ancient Egypt,^1^ whose estimated mean age at the time of death was only 45 years.

If the so-called “classical risk factors” were less prevalent in ancient times, different, non-traditional factors must have played a significant role in the development and progression of atherosclerosis.^2^ Microbial and parasitic inflammatory burdens that were likely present in ancient cultures inherently lacking modern hygiene and antimicrobials could have evoked a chronic inflammatory status. Given that patients with today’s chronic systemic inflammatory diseases, including human immunodeficiency virus infection, systemic lupus erythematosus, and rheumatoid arthritis experience early-onset atherosclerosis and coronary events, is it possible that the chronic inflammatory load secondary to infection resulted in atherosclerosis in ancient times? Moreover, atherosclerosis is a complex, multifactorial biological process, and, as such, it is also subject to gene-environment interplay; therefore, although the contribution of today’s classical risk factors to the development of atherosclerosis is inquestionable, their role in the appearance of atherosclerotic lesions in the vascular tree involves not only inflammation and activation of the immune system but also genetic factors that facilitate or oppose the formation of lipid accumulation in the arterial wall.

Progress in cell and molecular biology has allowed us to refine our understanding of the mechanisms involved in the onset of atherosclerosis. LDL-cholesterol particles play a significant role in the genesis of atherosclerotic plaque in the presence of endothelial dysfunction, an omnipresent feature in individuals with cardiovascular risk factors.^3^ Proliferation and migration of smooth muscle cells in response to the release of growth factors and the accumulation of mononuclear phagocytes rich in plasma-derived lipids (foam cells) contribute to the development of atheroma.^4^ Further studies revealed that the immune system played a role in atherosclerosis through not only innate (macrophages) but also adaptive (T cell and other lymphocytes) pathways.^5^ Cells directly involved in atherosclerosis establish a complex network of cross-talking by the release of cytokines, notably interleukin-1.^6^

Once recognized as an inflammatory disease, a highly sensitive assay for the measurement of C-reactive protein (hsCRP) proved to be a marker for patients at high risk for cardiovascular events due to atherosclerosis and a useful tool in selecting patients for aggressive lipid control for risk reduction. In the JUPITER trial, statin therapy in patients with hsCRP values above the median for the population (> 2 mg/L) but with LDL-cholesterol level < 130 mg/dL had a 44% reduction in first-ever cardiovascular events.^7^ More recently, the CANTOS trial allocated the anti-interleukin-1 antibody (canakinumab) to patients with stable post-acute coronary syndromes who had hsCRP values > 2 mg/L on statin therapy.^8^ Individuals who achieved a reduction of hsCRP to < 2 mg/L in response to anti-inflammatory therapy had a > 30% reduction in cardiovascular and all-cause mortality.^9^

It is now widely accepted that genetic factors also contribute significantly to the risk of coronary artery disease (CAD), and the heritability of CAD has been estimated to be between 40% and 60%.^10^ Using genome-wide association studies (GWAS), common single nucleotide polymorphisms (SNP) present in ≥ 5% of the population across the human genome can be identified, and the allele frequency of each SNP can be compared in cases and controls. The first association of CAD revealed by the GWAS approach was a block containing multiple SNP at 9p21.3 locus.^11^ Since then, new SNP have been found to correlate significantly with the presence and extension of atherosclerotic CAD; these SNP are related to different aspects that govern the biology of atherosclerosis including, but not limited to, cell-to-cell interactions, immune response, cholesterol absorption, and lipoprotein(a) levels.^12^ The CRP gene +1444C > T variant in the 3’ untranslated region (UTR) influences both basal and stimulated CRP levels.^13^

Investigators from different research facilities have produced a complex, most likely still incomplete picture of the intricate relationship between inflammation-infection, genetics, and atherosclerotic diseases like CAD. In this issue of the *Brazilian Archives of Cardiology*, Rocha et al.,^14^ sought to investigate the link between periodontal disease (as a model of chronic inflammatory condition) and two specific polymorphisms in genes knowingly related to inflammation (C-reactive protein and interleukin-6), with the presence of CAD in 80 patients from the South Region of Brazil referred for invasive coronary angiography. They found in the multivariate model that male gender and the CRP gene +1444C > T variant were significantly associated with the presence of CAD.

The authors should be congratulated for their contribution to the field, because replicating the finding of a significant association between a specific SNP and clinical outcomes in different populations strengthens the finding’s relevance to the occurrence of the measured outcome. Nevertheless, the study would benefit from providing the levels of hsCRP in the population study (as a measurement of chronic inflammatory status) and further detailing the extension of the obstructive pattern, rather than only a dichotomous classification of CAD present/absent based on the medical report. Additionally, I would be very cautious in dismissing the possible association between the IL-6 gene variant and the presence of CAD because of the small number of patients included in the study, which yielded a marginal statistical significance in the multivariate model (p-value = 0.06), given that this association has been validated in previous studies mentioned by the authors.

Despite its limitations, the work by Rocha et al.^14^ draws clinical cardiologists’ attention to the fact that genetics has gone beyond the laboratory and is currently making its way into clinical practice, at least as a tool to help us better understand how different our patients are, even if they are exposed to the same risk factors with which we are all too familiar. We will have to learn how to apply genetic risk scores that may improve our ability to predict the risk of CAD^14^ beyond what is estimated based on traditional risk factors alone. Gregor Mendel would be delighted to see how far we have come from his early experiments with pea plants more than 150 years ago, but the road to fully understanding the role of genetic factors in complex diseases such as atherosclerosis or hypertension is a long, albeit fascinating one.
